# Supplementary material for: Biochemical and Transcriptome Analyses Reveal a Stronger Capacity for Photosynthate Accumulation in Low-Tillering Rice Varieties
Source: Int J Mol Sci. 2024 Jan 29;25(3):1648. doi: 10.3390/ijms25031648 (PMC10855222; doi:10.3390/ijms25031648)
Supplement: Supplementary file 1 [file ijms-25-01648-s001.zip › Figure S1.pdf]

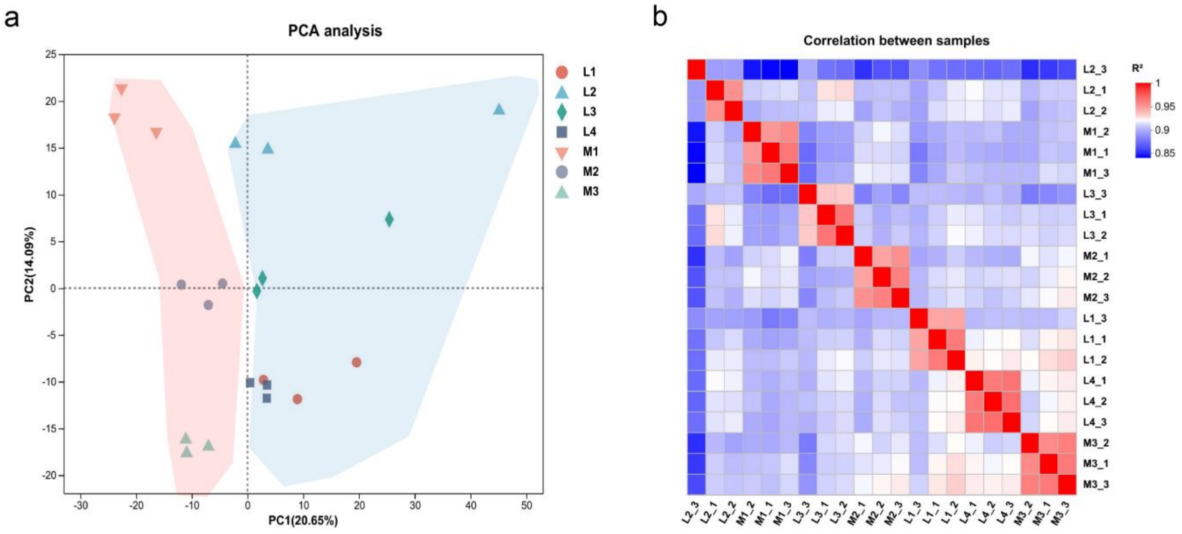

Figure S1: PCA analysis and correlation analysis between samples. (**a**) PCA analysis between samples. (**b**) Correlation analysis between samples. **Note**: L1: 9311PAY1, L2: V564, L3: R900, L4: R2257, M1: Yue 4B, M2: Di Gu, M3: Guichao 2 Hao.
