# Supplementary material for: Biochemical and Transcriptome Analyses Reveal a Stronger Capacity for Photosynthate Accumulation in Low-Tillering Rice Varieties
Source: Int J Mol Sci. 2024 Jan 29;25(3):1648. doi: 10.3390/ijms25031648 (PMC10855222; doi:10.3390/ijms25031648)
Supplement: Supplementary file 1 [file ijms-25-01648-s001.zip › Figure S3.pdf]

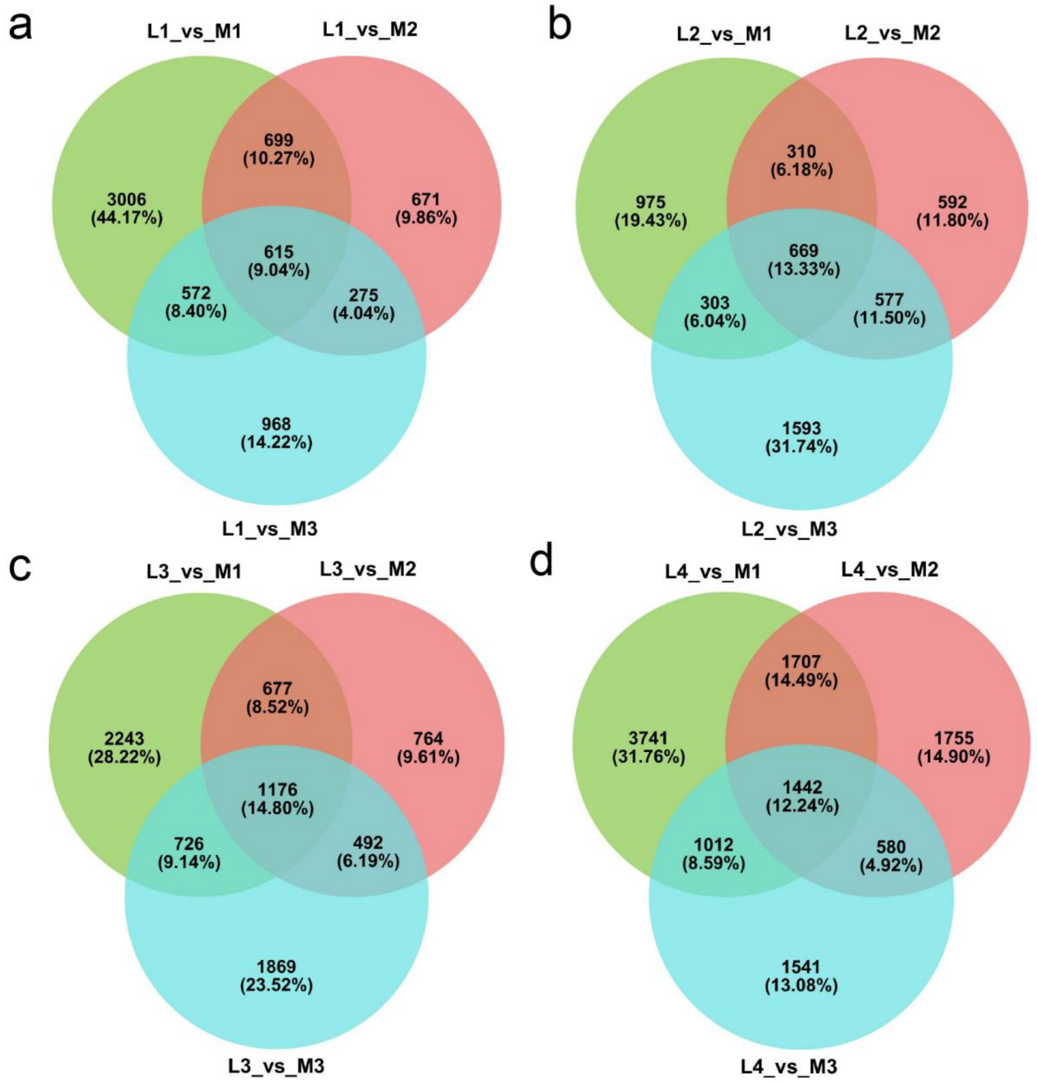

Figures S3 The venn analysis results of four low-tillering rice varieties and three multi-tillering rice varieties. (**a**) L1_vs_M: the common DEGs of L1_vs_M1, L1_vs_M2 and L1_vs_M3. (**b**) L2_vs_M: the common DEGs of L2_vs_M1, L2_vs_M2 and L2_vs_M3. (**c**) L3_vs_M: the common DEGs of L3_vs_M1, L3_vs_M2 and L3_vs_M3. (**d**) L4_vs_M: the common DEGs of L4_vs_M1, L4_vs_M2 and L4_vs_M3. **Note**: L1: 9311PAY1, L2: V564, L3: R900, L4: R2257, M1: Yue 4B, M2: Di Gu, M3: Guichao 2 Hao.
